# Supplementary material for: Diagnostic Performance of Circulating miRNAs and Extracellular Vesicles in Acute Ischemic Stroke
Source: Int J Mol Sci. 2022 Apr 20;23(9):4530. doi: 10.3390/ijms23094530 (PMC9102701; doi:10.3390/ijms23094530)
Supplement: Supplementary file 1 [file ijms-23-04530-s001.zip › ijms-1616774-supplementary.pdf]

Supplemental materials 1: MIFlowCyt-EV of clinical research study “Diagnostic Performance of Circulating miRNAs and Extracellular Vesicles in Acute Ischemic Stroke”

1 Flow cytometry

1.1 Experimental design

The aim of flow cytometry (A60-Micro, Apogee Flow Systems, Hemel Hempstead, UK) was to compare the concentrations of extracellular vesicles (EVs) released from platelets ($CD61^+$), activated platelets ($CD61^+/CD62P^+$), leukocytes ($CD45^+$) and endothelial cells ($CD146^+$) in platelet-depleted plasma (PDP) between patients at day 1 and day 7 after acute ischemic stroke, with and without high platelet reactivity (HPR), and control patients. We hypothesised that (1) patients at day 1 after acute ischemic stroke have higher EV concentrations (all types) than patients at day 7 after acute ischemic stroke and controls, and (2) patients at day 1 after acute ischemic stroke with HPR have higher concentrations of EVs derived from platelets, activated platelets, and leukocytes than patients with low platelet reactivity of patients at day 7 after acute ischemic stroke.

All samples were measured using an autosampler, which facilitates subsequent measurements of samples in a 96-well plate. The entire study involved four 96-well plates that were measured at August 28th 2018, August 31st 2018, October 16th 2021, and October 17th 2021. Each well plate contained buffer-only controls, antibody in buffer controls, and isotype controls. Flow rate and scatter calibrations were performed daily. Fluorescence calibrations were cross calibrated to a calibration in 2018 and 2021 using Sphero Rainbow Calibration Particles (RCP-30-5A), Spherotech, USA. To automatically determine optimal samples dilutions, apply calibrations, determine and apply gates, generate reports with scatter plots and generate data summaries, we used custom-built software (MATLAB R2018b, Mathworks, Natick, MA, USA).

1.2 Sample dilutions

Because the particle concentration in PDP differs between individuals, samples require different dilutions to avoid swarm detection [1] and to achieve statistically significant counts within a clinically applicable measurement time. Although serial dilutions are recommended

to find the optimal dilution, we consider serial dilutions unfeasible in a study with 63 donors. Therefore, we developed a procedure to estimate to optimal sample dilution (Section 1.2 of https://doi.org/10.6084/m9.figshare.c.4753676). In sum, we showed that for our flow cytometer and settings used, a count rate $\leq 5.0 \cdot 10^3$ events second unlikely results in swarm detection.

To find the dilution resulting in a count rate $\leq 5.0 \cdot 10^3$ events per second, we diluted each PDP sample 200-fold in citrated (0.32%), filtered (Whatman 50 nm, Sigma-Aldrich, US) phosphate buffered saline (PBS; samples 2018) or Dulbecco phosphate buffered saline (DPBS; samples 2021) and measured the total concentration of particles for 30 seconds without staining. Samples having a count rate $> 5.0 \cdot 10^3$ following 200-fold dilution were diluted 2,000-fold and measured. During the study, we simplified the pre-analytical part by switching to DPBS, which does not require filtering and pH adjustment.

Taking into account the measured total concentration of particles and flow rate, we calculated the minimum dilution required before staining (next section) to achieve a count rate $\leq 5.0 \cdot 10^3$ events per second after staining. The staining procedure adds an extra dilution of 11.3-fold to the overall dilution. To simplify procedures, samples were divided into 10 categories of pre-staining dilution: 10-fold, 16-fold, 25-fold, 40-fold, 50-fold, 65-fold, 100-fold, 150-fold, 160-fold, 260-fold.

1.3 EV staining

EVs in PDP were stained with antibodies. Prior to staining, antibodies were diluted in citrated (0.32%) PBS or DPBS and centrifuged at 18,890 g for 5 min to remove aggregates. Table S1 shows an overview of the used reagents and antibody concentrations during staining. Each sample was double labelled with CD45-APC (allophycocyanin) and CD146-PE (phycoerythrin), and with CD61-APC and CD62p-PE. To stain, 20 μL of 50-fold or 100-fold pre-diluted PDP was incubated with 2·2.5 μL of antibodies or isotype controls and kept in the dark for 2 h at room temperature. Post-staining, samples were diluted 11.3-fold in 200 μL of citrated (0.32%) PBS or DPBS to decrease background fluorescence from unbound reagents.

1.4 Buffer-only control

Each 96-wellplate contained at least 1 well with filtered (Whatman 50 nm) PBS or DPBS, which was measured with the same flow cytometer and acquisition settings as all other samples. The mean count rate was 21.5 events per second, which is substantially lower than the target count rate ($2.5\text{-}5.0 \cdot 10^3$ events per second) for PDP samples.

1.5 Buffer with reagents control

Each 96-wellplate contained a buffer with reagent control for each reagent (Table S1), which was measured with the same flow cytometer and acquisition settings as all other samples. CD45-APC, CD62p-PE, and CD146-PE in buffer resulted in 7, 0, and 4 fluorescence positive events, respectively. CD61-APC resulted in 124 fluorescence positive events, which is relatively high but substantially lower than the number of positive CD61-APC events in donor samples (752 on average).

1.6 Unstained controls

Unstained controls were not measured, because we never use them in our analysis.

1.7 Isotype controls

Table S1 shows an overview of the used isotype controls. For all isotype controls, the count rate of the buffer with isotype control antibodies was lower than the count rate of the buffer-only control. For particles with a diameter >200 nm and a refractive index <1.42, as reported in this study, we measured 0 APC+ events for IgG-APC and 0 PE+ events for IgG-PE during 120 seconds.

1.8 Trigger channel and threshold

Based on the buffer-only control (21.5 events s^{-1}), the acquisition software was set up to trigger at 14 arbitrary units SSC, which is equivalent to a side scattering cross section of 10 nm^2 (Rosetta Calibration, v1.23, Exometry, Amsterdam, The Netherlands).

1.9 Flow rate quantification

The A60-Micro is equipped with a syringe pump with volumetric control, which is checked on a daily basis using quality control beads and software (Apogee Flow Systems). In practice, the measured flow rate deviates at maximum 14% from the adjusted flow rate [2]. Therefore, we assumed that the flow rate is equal to the adjusted flow rate of 3.01 µL/min for all measurements.

1.10 Fluorescence calibration

Calibration of the fluorescence detectors from arbitrary units (a.u.) to molecules of equivalent soluble fluorochrome (MESF) was accomplished using 2 µm Q-APC beads (2321-175, BD) and SPHERO Easy Calibration Fluorescent Particles (ECFP-F2-5K, Spherotech Inc., Irma Lee Circle, IL, USA).

For each measurement, we added fluorescent intensities in MESF to the flow cytometry data files by custom-build software (MATLAB R2018a) using following equation:

$\mathrm{I(MESF)} = 10^{a\cdot\log_{10}\mathrm{I(a.u.)}+b}$	Equation S1

where I is the fluorescence intensity, and *a* and *b* are the slope and the intercept of the linear fits in figures S1A-D, respectively.

1.11 Light scatter calibration

We used Rosetta Calibration to relate scatter measured by FSC or SSC to the scattering cross section and diameter of EVs. Figure S2 shows print screens of the scatter calibrations. We modelled EVs as core-shell particles with a core refractive index of 1.38, a shell refractive index of 1.48, and a shell thickness of 6 nm. For each measurement, we added the FSC and SSC scattering cross sections and EV diameters to the flow cytometry datafiles by custom-build software (MATLAB R2018a). The SSC trigger threshold corresponds to a side scattering cross section of 10 nm^2.

1.12 EV diameter and refractive index approximation

Flow-SR was applied to determine the size and refractive index of particles and improve specificity by enabling label-free differentiation between EVs and lipoprotein particles [3,4]. Flow-SR was performed as previously described [3,4]. Lookup tables were calculated for diameters ranging from 10 to 1000 nm, with step sizes of 1 nm, and refractive indices from 1.35 to 1.80 with step sizes of 0.001. The diameter and refractive index of each particle was added to the .fcs file by custom-build software (MATLAB R2018a).

Because Flow-SR requires accurate measurements of both FSC and SSC, we applied Flow-SR only to particles with diameters >200 nm and fulfilling the condition:

$\mathrm{SSC(nm^2)} > -0.7\cdot\mathrm{FSC(nm^2)} + 3.2$	Equation S2

1.13 MIFlowCyt checklist

The MIFlowCyt checklist is added to Table S2.

1.14 EV number concentration

The concentrations reported in the manuscript describe the number of particles (1) that exceeded the SSC threshold, corresponding to a side scattering cross section of 10 nm^2, (2) that were collected during time intervals, for which the count rate was within 750 events/s from the median count rate, (3) with a diameter >200 nm as determined by Flow-SR [3], (4)

fulfilling the condition of equation S2, (5) having a refractive index <1.42 to omit false positively labeled chylomicrons, and (6) are positive at the corresponding fluorescence detector(s), per mL of PDP.

For the samples stained with CD61-APC and CD62-PE, two extra gates were applied between aforementioned steps 2 and 3. To omit residual platelets, only events with a side scattering cross section <1,500 nm^2 and an APC intensity <5,000 MESF were included. To omit CD61 aggregates, only events fulfilling the condition:

$\mathrm{SSC}(\mathrm{nm}^2) > 1.0 \cdot \mathrm{APC}(\mathrm{MESF}) - 1.4$	Equation S3

were included. For the samples stained with CD45-APC and CD146-PE, one extra gate was applied between aforementioned steps 2 and 3. To omit residual platelets, only events with a side scattering cross section <1,500 nm^2 were included.

1.15 Data sharing

Raw data, data with standard units, and a summary of all flow cytometry scatter plots and gates applied will be shared upon request.

2 References

[1] van der Pol E, van Gemert MJC, Sturk A, Nieuwland R, van Leeuwen TG. Single vs. swarm detection of microparticles and exosomes by flow cytometry. *J Thromb Haemost* 2012; **10**: 919–30.

[2] Gasecka A, Nieuwland R, Budnik M, Dignat-George F, Eyileten C, Harrison P, Lacroix R, Leroyer A, Opolski G, Pluta K, van der Pol E, Postuła M, Siljander P, Siller-Matula JM, Filipiak KJ, others. Ticagrelor attenuates the increase of extracellular vesicle concentrations in plasma after acute myocardial infarction compared to clopidogrel. *J Thromb Haemostaris* 2020; **18**: 609–23.

[3] van der Pol E, de Rond L, Coumans FAW, Gool EL, Böing AN, Sturk A, Nieuwland R, van Leeuwen TG. Absolute sizing and label-free identification of extracellular vesicles by flow cytometry. *Nanomed Nanotechnol Biol Med* 2018; **14**: 801–10.

[4] de Rond L, Libregts SFWM, Rikkert LG, Hau CM, van der Pol E, Nieuwland R, van Leeuwen TG, Coumans FAW. Refractive index to evaluate staining specificity of extracellular vesicles by flow cytometry. *J Extracell Vesicles* 2019; **8**: 1643671.

Figures

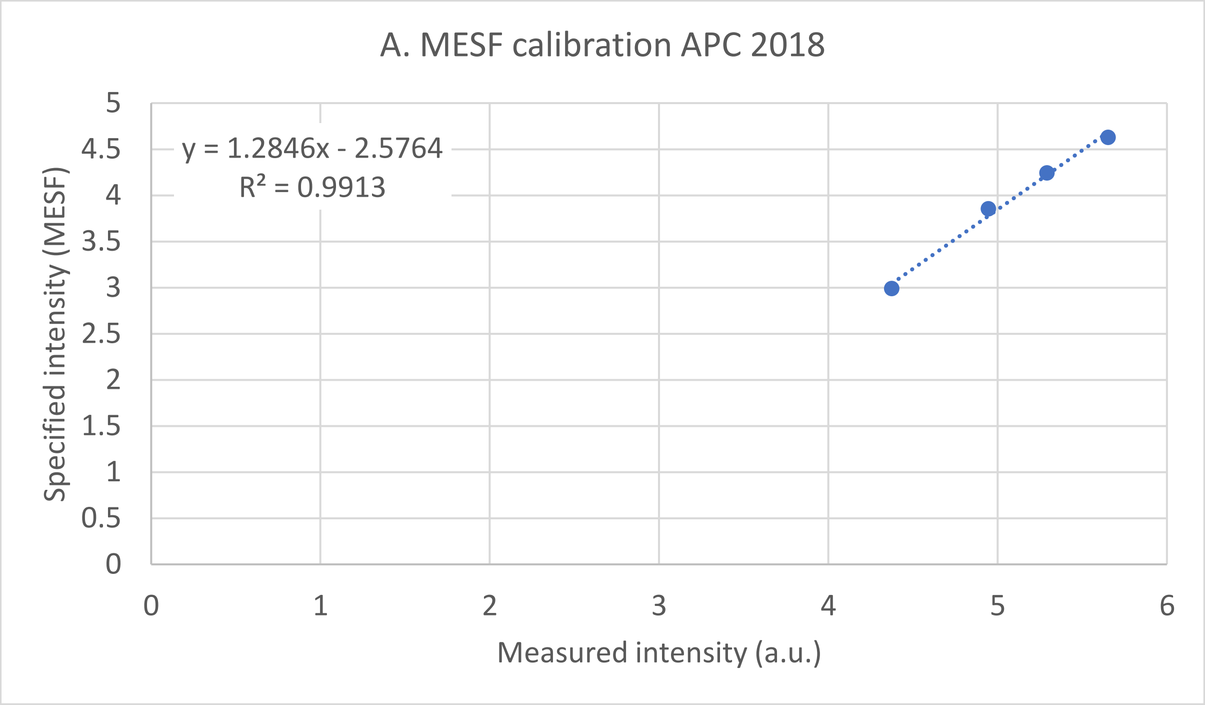
A. MESF calibration APC 2018
y = 1.2846x - 2.5764
R² = 0.9913
Specified intensity (MESF)
Measured intensity (a.u.)
0
0.5
1
1.5
2
2.5
3
3.5
4
4.5
5
0
1
2
3
4
5
6

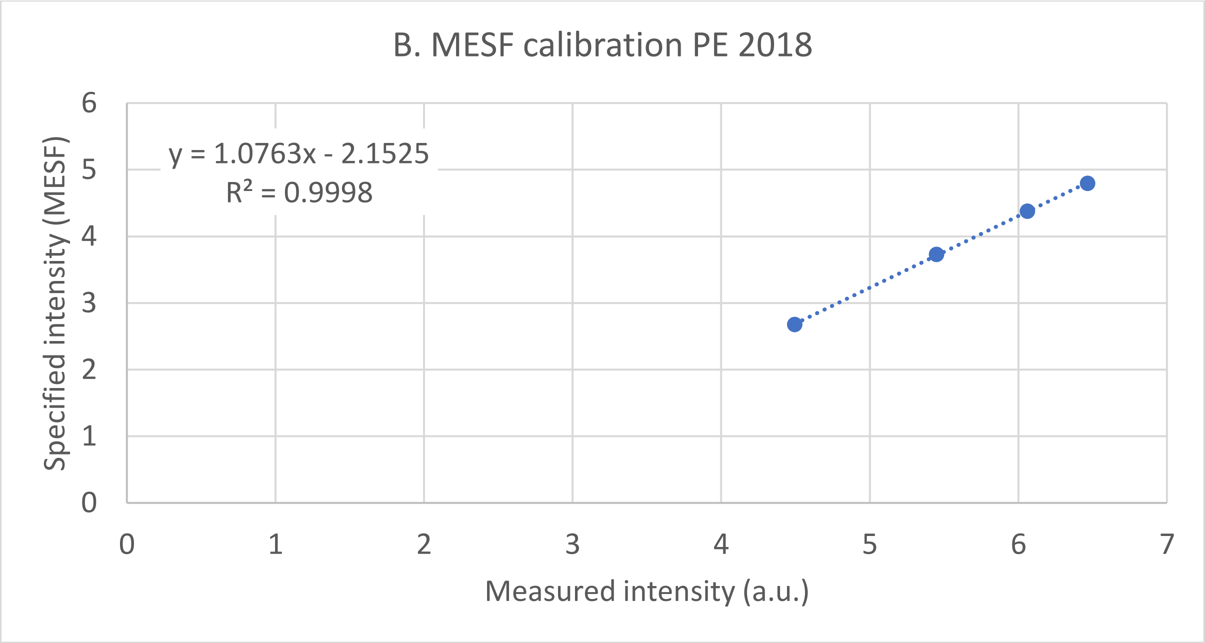
B. MESF calibration PE 2018
y = 1.0763x - 2.1525
R² = 0.9998
Specified intensity (MESF)
Measured intensity (a.u.)
0
1
2
3
4
5
6
0
1
2
3
4
5
6
7

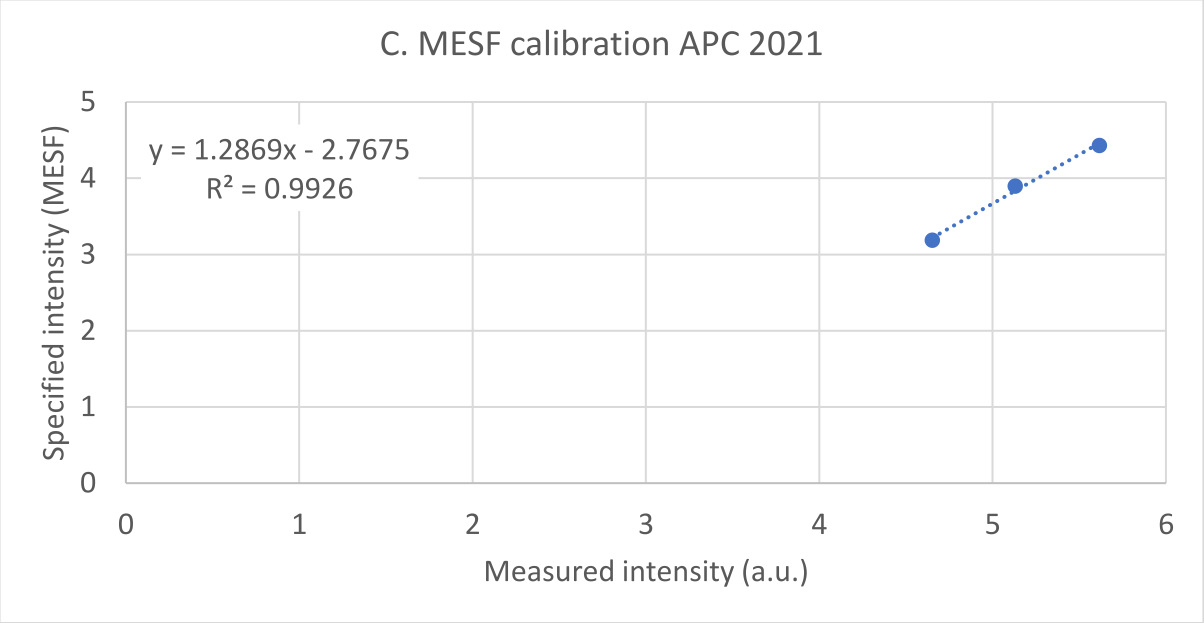

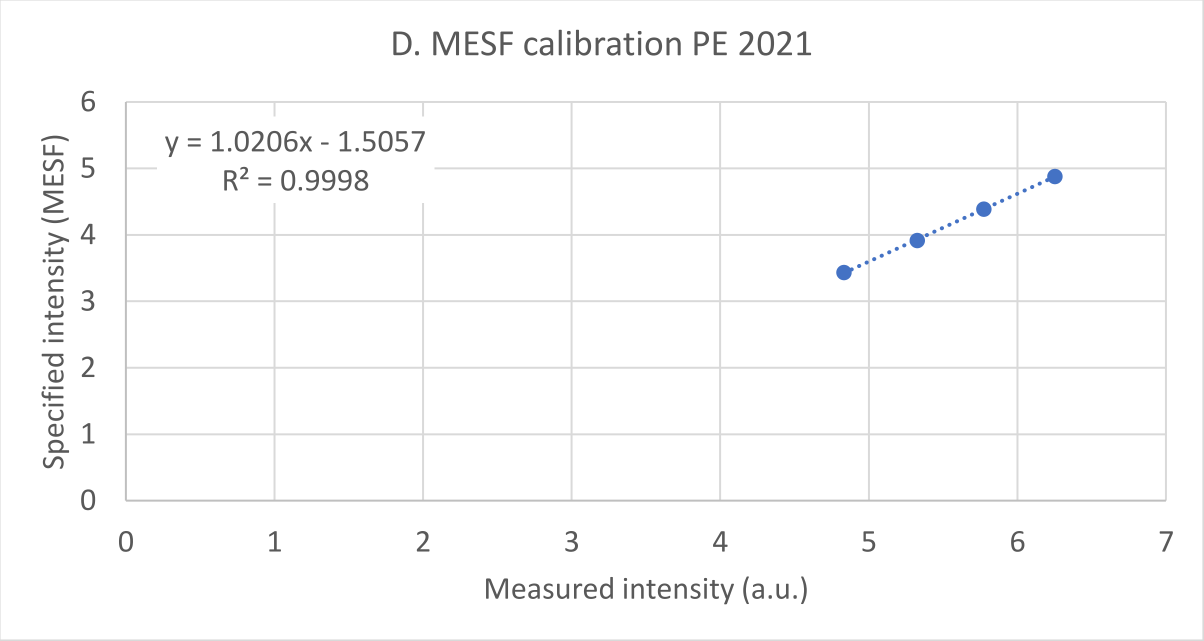

Figure S1. Calibration of the fluorescence detectors from arbitrary units (a.u.) to molecules of equivalent soluble fluorochrome (MESF). Logarithmic MESF versus logarithmic mean fluorescence intensity (MFI) for (A) APC in 2018, (B) phycoerythrin (PE) in 2018, APC in 2021 and PE in 2021. Data (symbols) are fitted with a linear function (line).

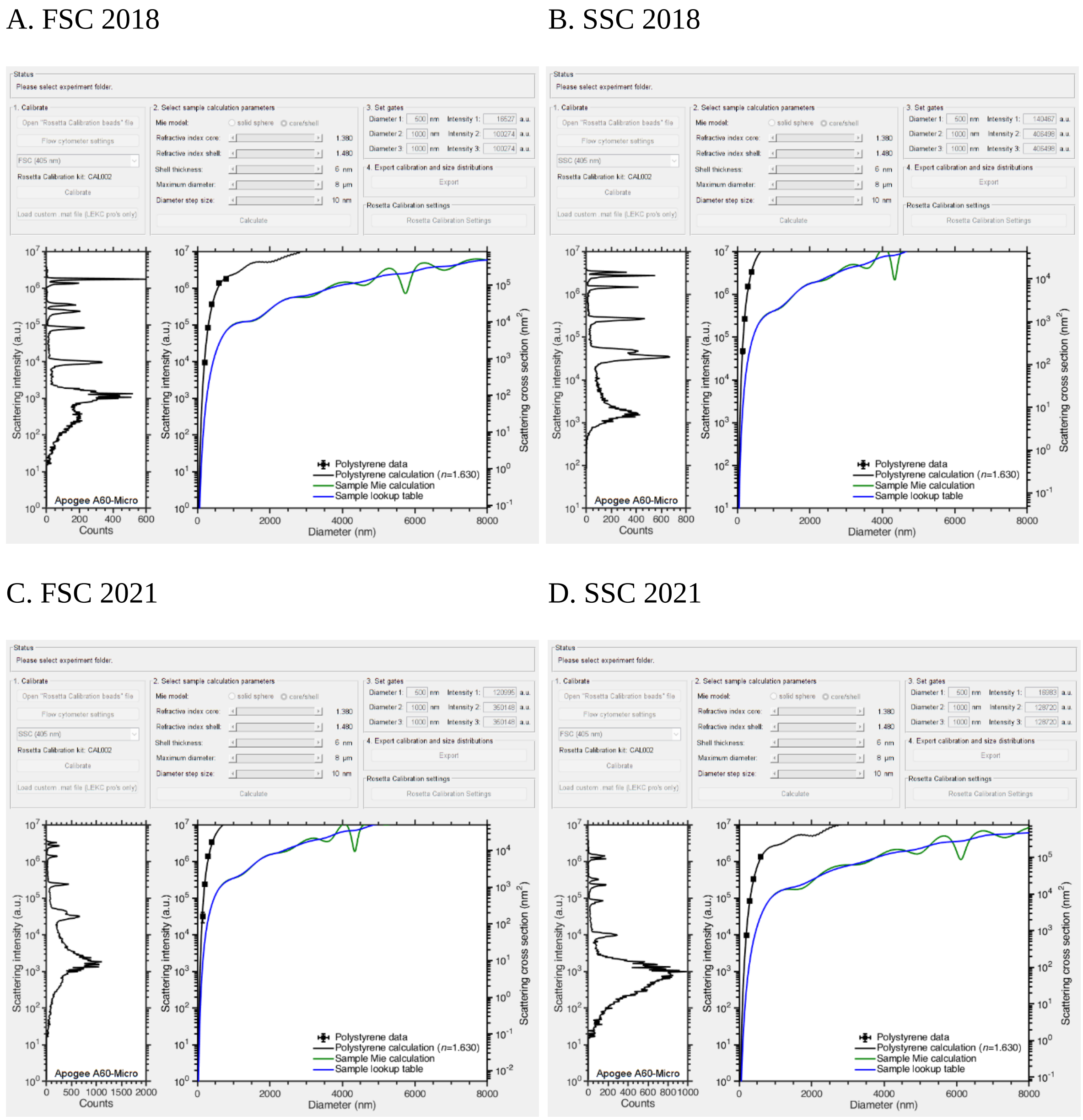

Figure S2. Forward scatter and side scatter calibration of the A60-Micro by Rosetta Calibration in 2018 and 2021. To relate scatter to the diameter of EVs, we modelled EVs as core-shell particles with a core refractive index of 1.38, a shell refractive index of 1.48, and a shell thickness of 6 nm.

Table S1: Overview of staining reagents. Characteristics being measured, analyte, analyte detector, reporter, isotype, clone, concentration, manufacturer, catalog number and lot number of used staining reagents. The antibody concentration during measurements was 11.3-fold lower than the antibody concentration during staining.

**Characteristic measured**	**Analyte**	**Analyte detector**	**Reporter**	**Isotype**	**Clone**	**Concentration during staining (μg mL^{-1})**	**Manufacturer**	**Catalog number**	**Lot number**
Integrin	Human CD61	Anti-human CD61 antibody	APC	IgG1	Y2/51	50	Dako	C7280	200483345
Adhesion molecule	Human CD62p	Anti-human CD62P antibody	PE	IgG1	CLB Thromb/6	6.25	Beckman Coulter	IM1759U	37
Leukocyte common antigen	CD45	Anti-human CD45 antibody	APC	IgG1	2D1	25	Beckman Dickinson	340910	5040555
Adhesion molecule	CD146	Anti-human CD146 antibody	PE	IgG1	S-Endo 1	3.75	Biocytex	5050-PE100T	173455
Affinity for Fc receptor	Fc receptor	IgG1	APC	n.a.	X40	200	Beckman Dickinson	554681	7075605
	Fc receptor	IgG1	PE	n.a.	IS5-21F5	50	Beckman Dickinson	345816	7248665

APC: allophycocyanin; CD: cluster of differentiation; IgG: immunoglobulin G; PE: phycoerythrin.

Table S2. MIFlowCyt checklist.

Requirement	Please Include Requested Information
1.1. Purpose	See section 1.1.
1.2. Keywords	miRNA, microRNA, microvesicles, extracellular vesicles, platelet, antiplatelet therapy, platelet reactivity, ischemia, stroke
1.3. Experiment variables	Absence or presence of acute ischemic stroke, time after acute ischemic stroke, platelet reactivity.
1.4. Organization name and address	Amsterdam University Medical Centers<br>Location Academic Medical Centre<br>Meibergdreef 9<br>1105 AZ Amsterdam<br>The Netherlands
1.5. Primary contact name and email address	Aleksandra Gąsecka, a.gasecka@amsterdamumc.nl
1.6. Date or time period of experiment	August 2018 to October 2021
1.7. Conclusions	Increased platelet activation in acute ischemic stroke is accompanied by elevated platelets- and leukocytes-derived EVs in the first 24 hours after ischemic episode, which decrease to baseline after 7 days. Patients with acute ischemic stroke had significantly higher platelet-EVs (CD61+) concentration both at day-1 and day-7 post-stroke compared to control patients. Similarly, leukocyte-EVs (CD45+) was significantly higher in acute ischemic stroke patients at day-1 compared to the control group. These results show the diagnostic ability of both platelet- and leukocyte-EVs in acute ischemic stroke. Importantly, pooling platelet-EVs and leukocyte-EVs together improved the accuracy of diagnostic utility than the value of each individual biomarker.
1.8. Quality control measures	All samples were measured using an autosampler, which facilitates subsequent measurements of samples in a 96-well plate (see MIFlowCyt 1.9). Each well plate contained buffer-only controls (section S1.4), antibody in buffer controls (section S1.5), unstained controls (section S1.6) and isotype controls (section

	S1.7). The flow rate was checked with ApoCal beads and software (Apogee Flow Systems). Fluorescence detectors were calibrated (section S1.10) with 2 µm Q-APC beads (2321-175, BD) and SPHERO Easy Calibration Fluorescent Particles (ECFP-F2-5K, Spherotech Inc., Irma Lee Circle, IL, USA). FSC and SSC were calibrated with Rosetta Calibration (v1.23, section S1.11).
1.9 Other relevant experiment information	The entire study involved four 96-well plates that were measured during 4 days.
2.1.1.1. Sample description	Thawed PDP (MIFlowCyt 2.1.1.2) from hospitalized humans after acute ischemic stroke and control patients (MIFlowCyt 2.1.1.3).
2.1.1.2. Biological sample source description	Venous blood samples were collected from the control group and patients with ischemic stroke on acetylsalicylic acid therapy (a) 24 hours after onset of acute ischemic stroke, (b) 7-days following index hospitalization. Briefly, blood was collected in 10-mL citrated blood collection tubes (S-Monovette, Sarstedt) via antecubital vein puncture using a 19-gauge needle, without tourniquet. The first 2 mL were discarded to avoid pre-activation of platelets. Right after blood collection, PDP was prepared by double centrifugation. Supernatant PDP was transferred into 1.5 mL low-protein binding Eppendorfs (Thermo Fisher Scientific) tubes, and stored in −80°C until analyzed. At each time point during blood sampling, an additional blood sample was collected to a 2.7-mL hirudin tube (S-Monovette, Sarstedt) to assess platelet function by using multiple electrode aggregometry (MEA, Roche Diagnostics). Platelet activity analysis was performed only once in the control group and twice in the ischemic stroke group (24 hours and 7-day post-stroke).
2.1.1.3. Biological sample source organism description	Hospitalized humans after acute ischemic stroke and control patients (for inclusion criteria, please see section 2.1 of the manuscript).
2.2 Sample characteristics	PDP is expected to contain erythrocyte ghosts, EVs, lipoproteins, proteins, and residual platelets.

2.3. Sample treatment description	Please see section S1.3.
2.4. Fluorescence reagent(s) description	Please see Table S1.
3.1. Instrument manufacturer	Apogee, Hemel Hempstead, UK
3.2. Instrument model	A60-Micro
3.3. Instrument configuration and settings	Samples were analysed for 120 seconds at a flow rate of 3.01 μL/min on an A60-Micro, equipped with a 405 nm laser (100 mW), 488 nm laser (100 mW) and 638 nm laser (75 mW). The trigger threshold was set at SSC 14 arbitrary units, corresponding to a side scattering cross section of 10 nm^2 (Rosetta Calibration). For FSC and SSC, the PMT voltages were 380 V and 360 V, respectively, in 2018. For FSC and SSC, the PMT voltages were 348 V and 350 V, respectively, in 2021. APC signals were collected with the 638-D Red(Peak) detector (long pass 652 nm filter, PMT voltage 510 V in 2018 and 423 V in 2021). PE signals were collected with the 488-Orange(Peak) detector (575/30 nm band pass filter, PMT voltage 520 V in 2018 and 459 V in 2021).
4.1. List-mode data files	Raw data, data with standard units and a summary of all flow cytometry scatter plots and gates applied are available upon request.
4.2. Compensation description	No compensation was required because no fluorophore combinations were used that have overlapping emission spectra.
4.3. Data transformation details	No data transforms were applied besides calibrations.
4.4.1. Gate description	See section 1.14
4.4.2. Gate statistics	The number of positive events was corrected for flow rate, measurement time and dilutions performed during sample preparation.
4.4.3. Gate boundaries	On overview of all gates is available upon request.

CD: cluster of differentiation; EVs: extracellular vesicles; FSC: forward scattering; PDP: platelet free plasma; SSC: side scattering.
